# Magnetite Nanoparticles Functionalized with Therapeutic Agents for Enhanced ENT Antimicrobial Properties

**DOI:** 10.3390/antibiotics11050623

**Published:** 2022-05-05

**Authors:** Mara Caciandone, Adelina-Gabriela Niculescu, Valentina Grumezescu, Alexandra Cătălina Bîrcă, Ionuț Cosmin Ghica, Bogdan Ștefan Vasile, Ovidiu Oprea, Ionela Cristina Nica, Miruna Silvia Stan, Alina Maria Holban, Alexandru Mihai Grumezescu, Ion Anghel, Alina Georgiana Anghel

**Affiliations:** 1 “Carol Davila” University of Medicine and Pharmacy, 050474 Bucharest, Romania; mara.caciandone@yahoo.com (M.C.); ionangheldoc@yahoo.com (I.A.); dr_alina.anghel@yahoo.com (A.G.A.); 2 Department of Science and Engineering of Oxide Materials and Nanomaterials, Faculty of Applied Chemistry and Materials Science, Politehnica University of Bucharest, 011061 Bucharest, Romania; adelina.niculescu@upb.ro (A.-G.N.); ada_birca@yahoo.com (A.C.B.); ghicaionutcosmin@gmail.com (I.C.G.); bogdan.vasile@upb.ro (B.Ș.V.); 3 Lasers Department, National Institute for Lasers, Plasma and Radiation Physics, 077125 Magurele, Romania; valentina.grumezescu@inflpr.ro; 4 Department of Inorganic Chemistry, Physical Chemistry and Electrochemistry, Faculty of Applied Chemistry and Materials Science, Politehnica University of Bucharest, 011061 Bucharest, Romania; ovidiu.oprea@upb.ro; 5 Department of Biochemistry and Molecular Biology, Faculty of Biology, University of Bucharest, 050095 Bucharest, Romania; cristina.nica@drd.unibuc.ro (I.C.N.); miruna.stan@bio.unibuc.ro (M.S.S.); 6 Department of Microbiology and Immunology, Faculty of Biology, University of Bucharest, 077206 Bucharest, Romania; alina_m_h@yahoo.com; 7 Research Institute of the University of Bucharest—ICUB, University of Bucharest, 050657 Bucharest, Romania; 8 Academy of Romanian Scientists, Ilfov No. 3, 050044 Bucharest, Romania; 9 “Dr. Carol Davila” Central Military Emergency University Hospital, 010825 Bucharest, Romania; 10 ENT Department, Saint Mary Clinical Hospital Bucharest, 011172 Bucharest, Romania

**Keywords:** magnetite nanoparticles, biopolymeric spheres, antimicrobial delivery, human cells

## Abstract

In the context of inefficient antibiotics, antibacterial alternatives are urgently needed to stop the increasing resistance rates in pathogens. This study reports the fabrication and characterization of four promising magnetite-based antibiotic delivery systems for ENT (ear, nose and throat) applications. Magnetite nanoparticles were functionalized with streptomycin and neomycin and some were entrapped in polymeric spheres. The obtained nanomaterials are stable, with spherical morphology, their size ranging from ~2.8 to ~4.7 nm for antibiotic-coated magnetite nanoparticles, and from submicron sizes up to several microns for polymer-coated magnetite–antibiotic composites. Cell viability and antimicrobial tests demonstrated their biocompatibility on human diploid cells and their antibacterial effect against Gram-negative (*Pseudomonas aeruginosa*) and Gram-positive (*Staphylococcus aureus*) opportunistic bacteria. The presence of the polymeric coat proved an enhancement in biocompatibility and a slight reduction in the antimicrobial efficiency of the spheres. Our results support the idea that functional NPs and polymeric microsystems containing functional NPs could be tailored to achieve more biocompatibility or more antimicrobial effect, depending on the bioactive compounds they incorporate and their intended application.

## 1. Introduction

Bacterial infections represent a frequent cause of hospitalization worldwide, while nosocomial infections are common triggers of increased mortality and morbidity, especially within healthcare facilities [1,2,3]. Such infections could be prevented and treated using antibiotic therapeutics, as numerous antimicrobials have been developed over the years. However, antibiotic misuse and/or overuse has led to the emergence of antimicrobial resistance [4,5,6,7]. The development of bacterial resistance to conventional treatment options has resulted in the enhancement of microbial virulence, conferred on pathogens the ability to evade the immune system under biofilm protection, extended hospitalization times, and increased morbidity and mortality [7,8,9,10]. Thus, antimicrobial resistance represents a significant concern affecting modern healthcare, imposing substantial human and economic burdens and forcing us to find alternative treatment strategies [9,11,12,13,14].

Promising solutions to this global problem are envisaged through the advances in nanomedicine. The application of nanotechnology for medical purposes has revolutionized the diagnosis, monitoring, prevention, and treatment of diseases [15,16,17,18]. Of particular interest is the development of nanovehicles, for the controlled delivery of drugs, which can increase therapeutic effectiveness while diminishing toxicity towards healthy tissues [19]. Such improved pharmacotherapeutic outcomes are possible due to the advantageous properties of nanoparticles, including small size, high specific surface area, surface charge, solubility, drug loading ability, and intrinsic antibacterial effect, which could act synergically with classical antibiotics [9,15,20].

Magnetic nanoparticles have been widely studied in biomedicine, mainly because they can be controlled using an external magnetic field and minimum residual magnetism after removing it [5,21,22,23,24,25]. Moreover, magnetic nanoparticles (NPs) have low internal diffusion resistance and can damage bacteria by interfering with the thiol group at the respiratory base of these microorganisms [5,9,26]. In particular, magnetite-based nanostructures are considered attractive for developing unconventional antimicrobials that carry and release drugs in a controlled and targeted manner, while minimizing side effects due to the small amount of transported antibiotics [11,27,28,29,30,31]. However, one drawback of these structures is their unstable, oxidizing nature, which can be overcome through surface functionalization, as recent studies report [26,32,33,34].

An appealing strategy to protect magnetite nanoparticles from oxidizing is their incorporation into biopolymeric spheres, improving delivery characteristics and enhancing the system’s biocompatibility. Furthermore, polymers’ intrinsic physicochemical versatility and tunable functionality render them suitable for controlled and/or triggerable release mechanisms [16,35]. Poly(lactic-co-glycolic acid) (PLGA) is an FDA-approved choice for biomedical applications, which has demonstrated excellent biological behavior (safe, biocompatible, biodegradable), facile, and beneficial interactions with simple biologically active substances or complex macromolecules, easy hydrolyzing property inside the body, and adjustable compositional and microstructural features [6,11,36,37,38].

Chitosan (CS) is another polymer that proved useful in the design of nanostructured antimicrobials. CS has more than a protecting role, being also a biocompatibility enhancer, a drug carrier, and a potent antimicrobial agent [9,14,16,39,40,41]. Recently, it has been shown that nanoparticles entrapped in the CS network may switch the zeta-potential from negative to positive, thus, promoting cellular adhesion and retention of the delivery system at the target site [8,35].

Aminoglycoside antibiotics were demonstrated to be effective against Gram-positive and Gram-negative bacteria, and mycoplasma, being used in various pharmaceutical applications [42,43]. Nonetheless, their potent antimicrobial activity is overshadowed by the occurrence of toxic side effects, rendering them as last-resort antibiotics [44]. Thus, there alternative combined therapies towards overcoming this limitation must be sought.

Fe_3_O_4_, PLGA, and CS showed promising results in various synergistic therapies, such as controlled and triggered treatment of infections (e.g., *S. aureus, P. aeruginosa, C. albicans*), hyperthermia, and targeted delivery of anticancer drugs [13,16,37,45,46,47,48,49,50]. This study reports the fabrication of magnetite-based materials to deliver the antibiotics streptomycin and neomycin, embedded into biopolymeric spheres, namely PLGA-CS-Fe_3_O_4_@NEO and PLGA-CS-Fe_3_O_4_@STR.

## 2. Results

### 2.1. Physicochemical Characterization of Fe_3_O_4_-STR and Fe_3_O_4_-NEO Nanoparticles

**Figure 1:**
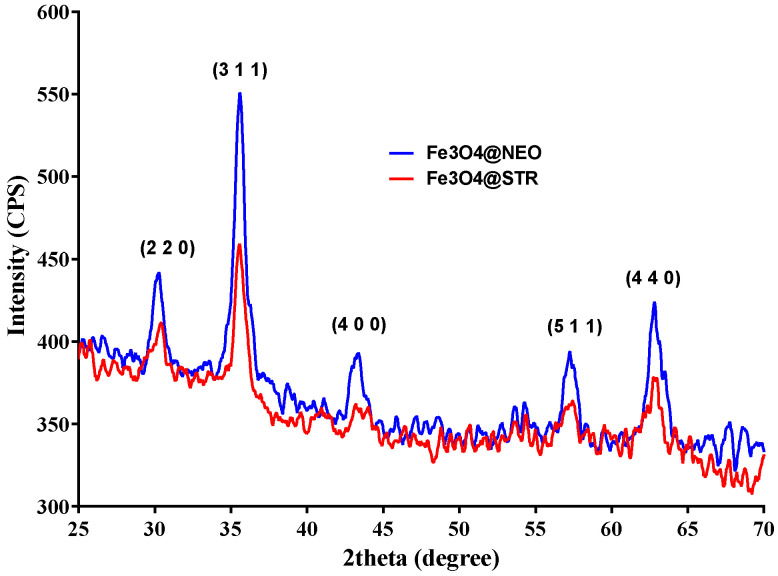
X-ray diffractogram of Fe_3_O_4_@STR and Fe_3_O_4_@NEO nanoparticles.

The XRD patterns of the antibiotic-functionalized magnetite nanoparticles are plotted in Figure 1. The diffraction peaks found at 2θ diffraction angles of 30.33°, 35.51°, 43.51°, 57.17°, and 62.5°, correspond to the diffraction planes (2 2 0), (3 1 1), (4 0 0), (5 1 1), and (4 4 0), respectively, being characteristic for crystalline magnetite with a spinel cubic structure. Thus, it is confirmed that the material’s crystallinity was not significantly affected by the addition of antibiotics. Comparing the results obtained for Fe_3_O_4_@STR and Fe_3_O_4_@NEO, a lower intensity was observed for the streptomycin-functionalized magnetite; nonetheless, this aspect does not affect the properties of the nanoparticles.

**Figure 2:**
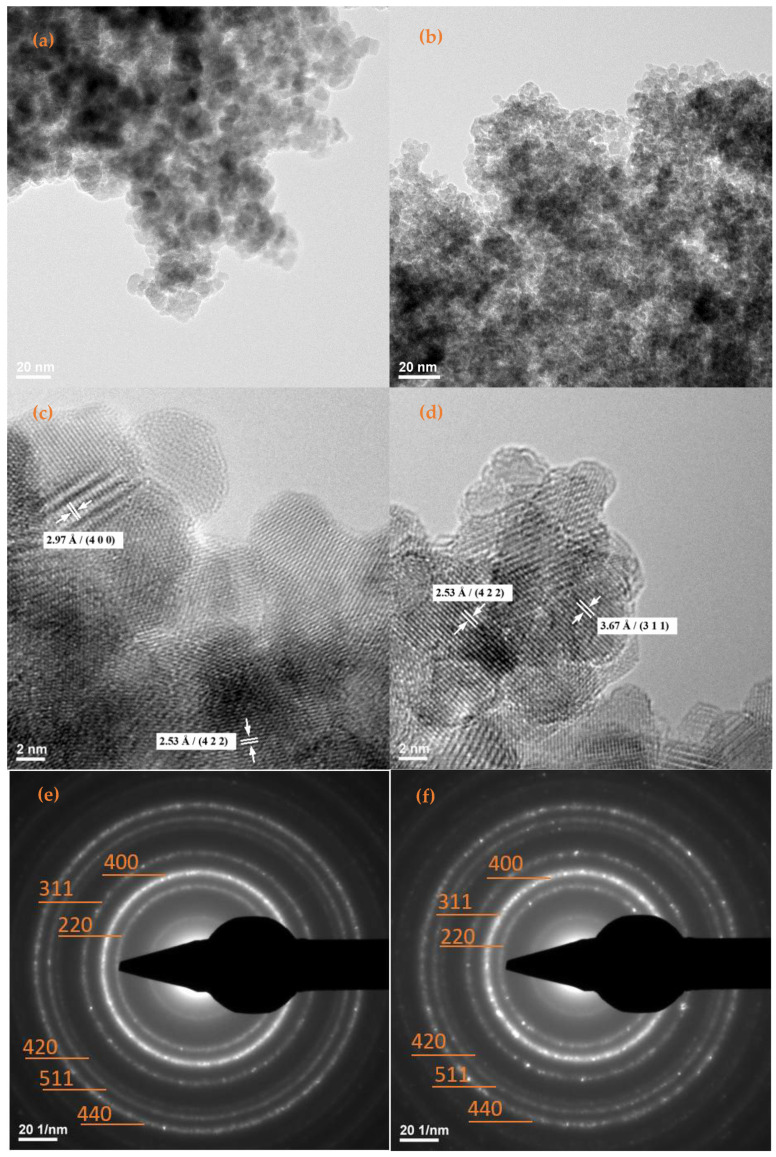

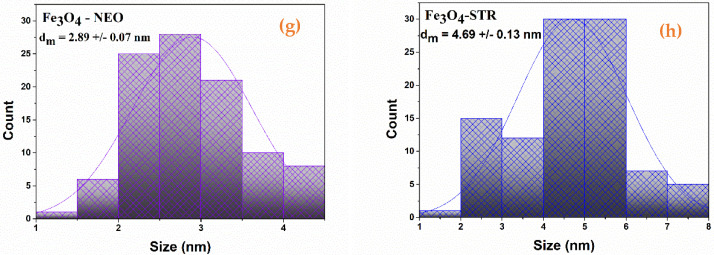
TEM images of (**a**,**c**) Fe_3_O_4_@NEO, (**b**,**d**) Fe_3_O_4_@STR; SAED patterns of (**e**) Fe_3_O_4_@NEO, (**f**) F_e3_O_4_@STR nanoparticles; histograms of (**g**) Fe_3_O_4_@NEO, (**h**) F_e3_O_4_@STR nanoparticles.

TEM and SAED analyses (Figure 2) allowed the determination of size, shape, aspect, growth direction, and nature of the formed crystalline nanoparticles. The TEM micrographs of Fe_3_O_4_@NEO (Figure 2a,b) and Fe_3_O_4_@STR (Figure 2d,e) nanoparticles, revealed that the quasi-spherical particles are covered by a non-crystalline layer (i.e., therapeutic agent) and have average dimensions of 2.89 ± 0.07 nm for Fe_3_O_4_@NEO and 4.690 ± 0.13 nm for Fe_3_O_4_@STR (Figure 2g,h).

Furthermore, Figure 2e,f display the concentric SAED rings of the functionalized magnetite nanoparticles formed at (220), (311), (400), (422), (511), and (440), which are in excellent agreement with the results obtained by XRD analysis. Thus, the presence of the same diffraction planes and the crystalline nature of the prepared materials are confirmed.

**Figure 3:**
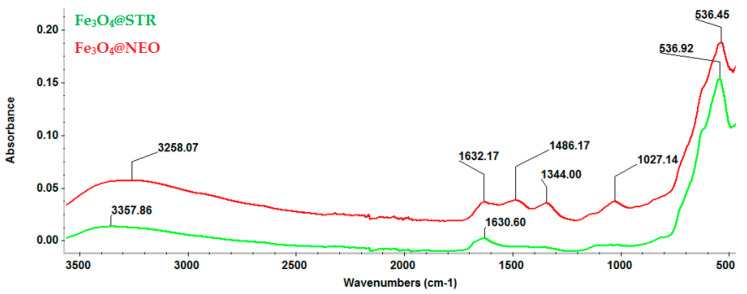
FT-IR spectra of Fe_3_O_4_@STR and Fe_3_O_4_@NEO nanoparticles.

FT-IR analysis (Figure 3) emphasized the integrity of functional groups characteristic of the synthesized particles. For both tested materials, a strong peak can be observed at 536 cm^−1^, attributed to Fe-O stretching vibrations from the structure of Fe_3_O_4_. The adsorption bands found between 1640 and 1300 cm^−1^ confirm the presence of the antibiotic drugs in the studied materials, being characteristic of their molecular fingerprint area. The peak recorded at 1027 cm^−1^ is specific for C-O-C bonds attributed to the therapeutic agents’ pyranose structure.

**Figure 4:**
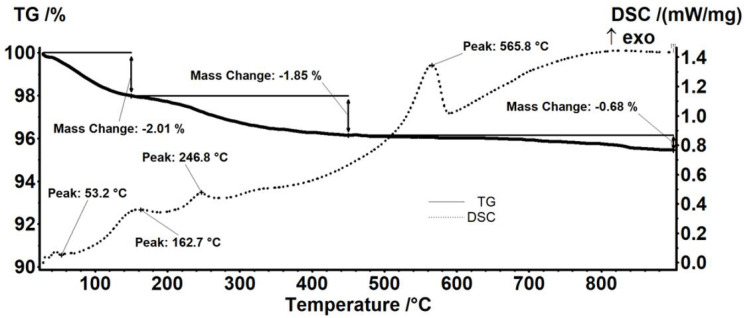
Thermogravimetric analysis of pristine Fe_3_O_4_ nanoparticles.

The thermogravimetric analysis of pristine Fe_3_O_4_ nanoparticles (Figure 4) indicated the presence of two mass loss steps. In the interval RT-150 °C, the magnetite sample loses 2.01% of its initial mass, and a weak endothermic effect accompanies the process on the DSC curve. This mass loss can be assigned to eliminating water molecules from the surface of nanoparticles. In the next step, between 150–450 °C, the recorded mass loss was of 1.85%, and a series of exothermic processes could be noticed. The mass loss is probably due to the elimination of -OH moieties from nanoparticles’ surfaces and the degradation of some impurities. The weak exothermic effect from 162.7 °C can be assigned to the oxidation of Fe^2+^ to Fe^3+^ (transformation of magnetite to maghemite) [51]. After 450 °C, the sample losed 0.68% of the initial mass. The intense exothermic peak from 565.8 °C is assigned to the phase transformation of maghemite to hematite [52].

**Figure 5:**
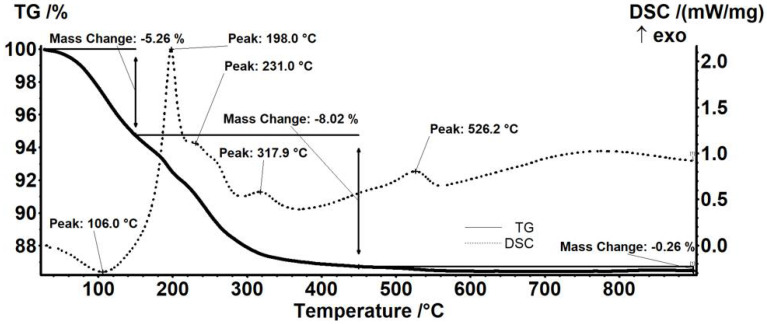
Thermogravimetric analysis of Fe_3_O_4_-NEO nanoparticles.

**Figure 6:**
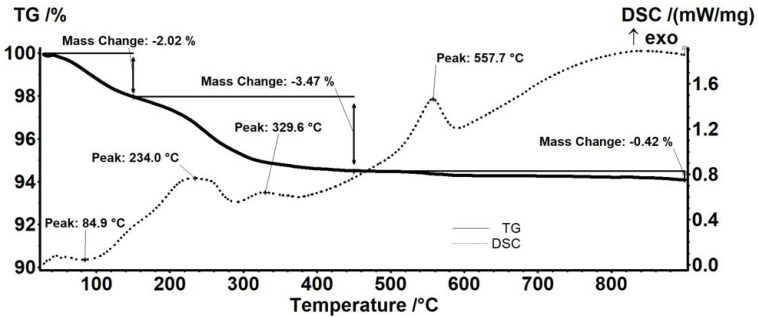
Thermogravimetric analysis of Fe_3_O_4_-STR nanoparticles.

The thermal analysis results showed differences in mass loss when comparing neomycin-functionalized (Figure 5) to streptomycin-functionalized (Figure 6) F_e3_O_4_ nanoparticles. The endothermic effect, with the minimum at 106.0 °C, is due to the physical elimination of water, a mass loss of 5.26% also being recorded up to 150 °C. The mass loss recorded in the 150–450 °C interval represents 8.02% of the initial mass. In this interval, the transformation of magnetite to maghemite occurs by oxidation of Fe^2+^ to Fe^3+^ [51] and the removal of the terminal –OH moieties from the nanoparticles’ surface after the oxidation of the organic layer. The thermal analysis allows the observance of successive partially overlapped stages of organic substance degradation. These oxidative degradations are marked by strong exothermic effects with peaks at 198.0, 231.0, and 317.9 °C. A mass loss of 0.26% was also recorded between 450–900 °C (Figure 6). The exothermic peak corresponding to the transformation of maghemite to hematite appears at a temperature of 526.2 °C [52].

Thermal analysis of the streptomycin-functionalized magnetite sample showed the first mass loss of 2.02%, between RT-150 °C, which corresponds to water traces elimination. This trend is similar to the results obtained for previous samples. The associated endothermic effect presents a minimum of 84.9 °C. In the next step, 150–450 °C, a mass loss of 3.47% was recorded. The process was accompanied by a series of exothermic effects, with peaks at 234.0 or 329.6 °C. Along with the transformation of magnetite to maghemite by oxidation of Fe^2+^ to Fe^3+^, the oxidative degradation of the organic molecules from the surface of the nanoparticles takes place. Between 450–900 °C, the sample loses 0.42%. At the temperature of 557.7 °C, the exothermic effect characteristic to the transformation of maghemite to hematite appears (Figure 6).

The data from thermal analyses are presented in Table 1. The estimated load for the nanoparticles is 9.42% for neomycin and 1.44% for streptomycin.

### 2.2. Physicochemical Characterization of Composites

**Figure 7:**
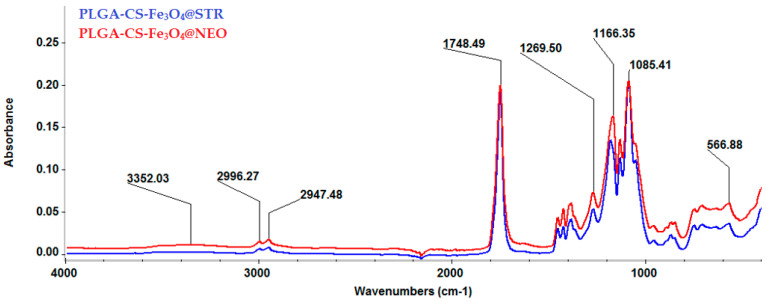
FT-IR spectra of PLGA-CS-Fe_3_O_4_@STR and PLGA-CS-Fe_3_O_4_@NEO spheres.

The IR spectra of the synthesized composite spheres are shown in Figure 7. For both types of materials, the absorption band was noticed at ~566 cm^−1^, which is characteristic for the Fe-O bonds stretching vibrations of magnetite core particles. However, as compared to the magnetite-based nanoparticles spectra, herein, the peak intensity was diminished due to the significant polymeric fraction present in the sample. The absorption bands from 2996 and 2947 cm^−1^ confirmed the presence of PLGA in the material’s composition, as these values are characteristic of C-H stretching and deformation vibrations from the polymer’s structure. The only absorption band identified and attributed to the presence of CS was found at 1085 cm^−1^, the rest being masked by the PLGA absorption bands. This peak is specific to C-O-C bonds that can be attributed to the glucosamine sequence from the structure of CS. The bands characteristic of the presence of antimicrobial therapeutic agents were also masked because of their low amounts in the sample compared to the amounts of polymers.

**Figure 8:**
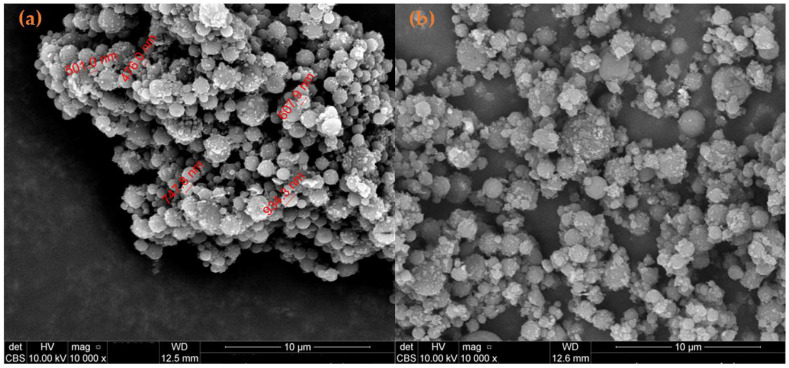
SEM images of (**a**) PLGA-CS-Fe_3_O_4_@NEO and (**b**) PLGA-CS-Fe_3_O_4_@STR composite spheres.

SEM micrographs (Figure 8a,b) of composite spheres showed a compact spherical shape. The diameter of the obtained spheres varies from submicron up to several microns, with an average of around 1 μm.

### 2.3. Biological Evaluation

#### 2.3.1. Cell Viability

**Figure 9:**
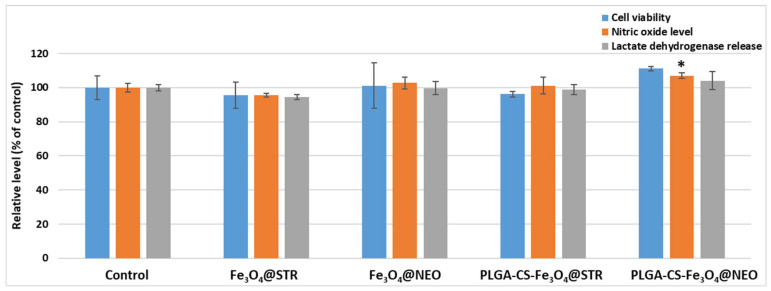
Biological evaluation of Fe_3_O_4_@STR and Fe_3_O_4_@NEO nanoparticles and PLGA-CS-Fe_3_O_4_@STR and PLGA-CS-Fe_3_O_4_@NEO spheres after 24 h of interaction with MRC-5 human cells by MTT test of cellular viability, NO level, and LDH release. The results were calculated as mean ± standard deviations of three different replicates and expressed relative to control cells (* *p* > 0.05 compared to control).

**Figure 10:**
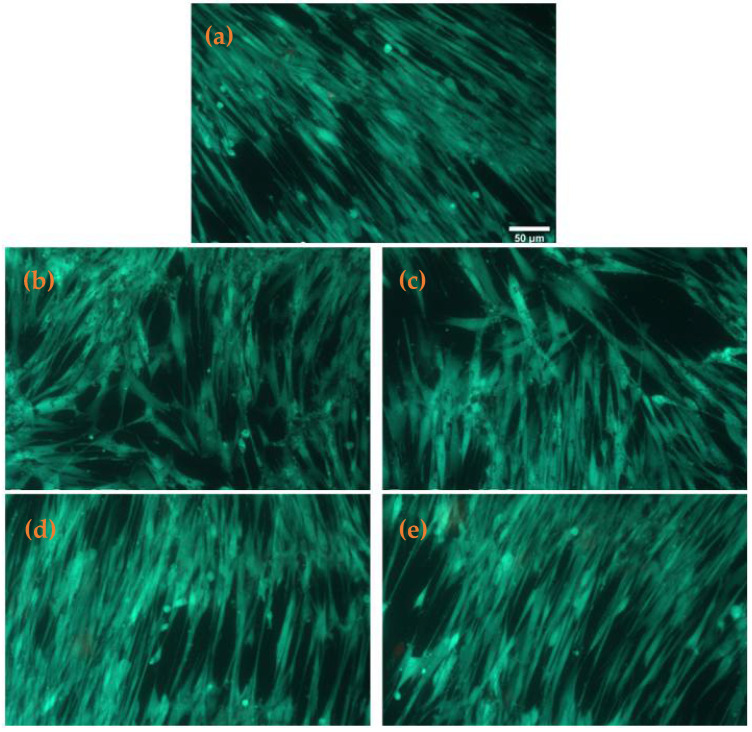
Fluorescence images of live (green) and dead (red) cells stained with calcein AM and ethidium, respectively, after 24 h incubation of MRC-5 human fibroblasts with (**a**) control, (**b**) Fe_3_O_4_@STR, (**c**) Fe_3_O_4_@NEO, (**d**) PLGA-CS-Fe_3_O_4_@STR and (**e**) PLGA-CS-Fe_3_O_4_@NEO spheres (scale bar is 50 µm and it is the same for all images).

For the biological characterization of the synthesized materials, the percent of metabolically active cells was evaluated after 24 h through the MTT assay on human lung fibroblasts (Figure 9). No significant changes were observed for the tested Fe_3_O_4_-STR and Fe_3_O_4_-NEO nanoparticles, as compared to the control sample, the level being 96% of untreated cells. An 11% increase in the cellular viability was noticed for PLGA/CS/PVA-Fe_3_O_4_-NEO microspheres, as compared to the untreated control. This effect is statistically significant, and it could be correlated with enhanced biocompatibility of the used polymer and its effect to stimulate cell proliferation. Further, the biocompatibility of these samples was confirmed by evaluating the levels of NO and LDH release (Figure 9). The results of the cells grown in the presence of functionalized nanoparticles were similar to the untreated control, after 24 h of incubation. These evaluations showed that the synthesized nanoparticles and microspheres induced no inflammation and the absence of any toxic effect on the cellular membrane integrity. Fluorescence staining of live and dead cells showed that PLGA/CS/PVA-Fe_3_O_4_-STR microspheres slightly modify the number of viable cells compared to control samples (Figure 10).

#### 2.3.2. Antimicrobial Tests

**Figure 11:**
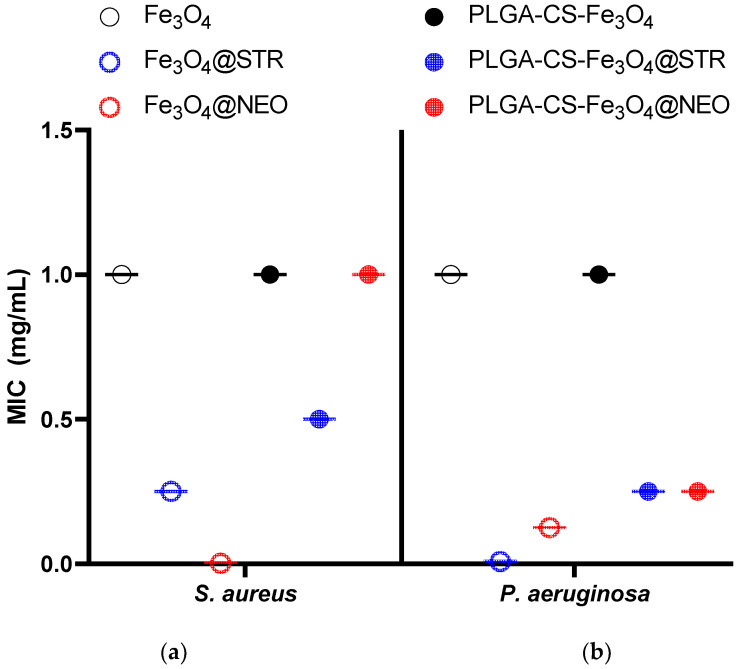
Minimum inhibitory concentration (MIC) of streptomycin- and neomycin-functionalized materials for (**a**) *S. aureus* and (**b**) *P. aeruginosa* at 24 h of incubation in standard conditions.

Antimicrobial effect was evaluated by a standardized test, aiming to establish the minimum inhibitory concentration (MIC) of antibiotic-functionalized nanomaterials against tested pathogens. The results, presented in Figure 11, revealed differences regarding MIC values of the obtained microspheres, depending on the embedded antibiotic and evaluated microbial species. The results demonstrated that microspheres containing streptomycin (STR) offer lower MIC values in *Staphylococcus aureus* and *Pseudomonas aeruginosa* evaluated strains. Our data suggest STR is better absorbed and released into the obtained nano and microsystem, considering the enhanced antimicrobial effect. Moreover, a microbial strain-dependent antibacterial efficiency was observed, as MIC values were twice lower in *P. aeruginosa,* for both STR and neomycin (NEO) containing NPs or polymeric microspheres, as compared to the S. *aureus* results (Figure 11). The polymeric coating was found to slightly reduce the antimicrobial efficiency of the magnetite-antibiotic nanosystem, as revealed by the MIC values for both microbial strains. This effect could correlate with improved biocompatibility in the polymeric microspheres, which could regulate the release of the bioactive compounds present in the functional NPs [53]. Moreover, this suggests that the bioactive agent and its release could be optimized in magnetite-PLGA-CS microspheres, to ensure a required biocompatibility and bioavailability, depending on the intended application [54,55].

The obtained results suggest that the synthesized materials have promising antibacterial inhibition properties that successfully design novel antimicrobial therapeutic delivery systems.

## 3. Discussion

The present study reported the fabrication of magnetite-based nanocomposites intended for antimicrobial drug delivery. Magnetite nanoparticles have been previously described in the literature as promising carriers for a wide range of antibiotics, including amoxicillin [56], cephalexin [57], ciprofloxacin [58], doxycycline [59], gentamicin [60], rifampicin [59], vancomycin [61], and more.

For this work, we opted to use magnetite-based nanoparticles to deliver aminoglycoside antibiotics, namely streptomycin and neomycin. These broad-spectrum antibiotics are recognized for their potent antimicrobial activity, even against drug-resistant bacterial strains [62]. For instance, Hu et al. [63] employed aminoglycosides against apramycin-resistant strains of *Escherichia coli* and *Klebsiella pneumoniae*, reporting promising susceptibility. More specifically, the authors obtained MIC_50_ and MIC_90_ values of 8 μg/mL and 256 μg/mL, respectively, for NEO, and 16 μg/mL and 256 μg/mL, respectively, for STR, against more than 65% of the tested carbapenem-resistant Enterobacteriaceae strains. Interesting results have also been described by Story and co-workers [64], who synthesized a series of pyrene-neomycin B conjugates towards enhancing their specificity and affinity while avoiding the resistance mechanisms of pathogens. Tests on the antimicrobials against Gram-positive bacteria were reported, and encouraging results were obtained, even towards methicillin-resistant *S. aureus* strains (MIC values between 12.5 and 50 μM).

In this work, the antimicrobial agents were prepared via the co-precipitation method, leading to quasi-spherical particles with an average size of 15 ± 1 nm. Similar patterns in size and morphology were observed in different studies employing the same synthesis technique. Mohammadi et al. [65] reported the synthesis of magnetite nanoparticles with diameters below 10 nm that were further coated with PEG, PEG-SiO_2_, and oleic acid. Similar results were recently reported by Ramadan et al. [66]. Two precipitating agents, namely sodium hydroxide and ammonium hydroxide were used, that led to the formation of Fe_3_O_4_ nanoparticles with an average size of 14 nm and 22 nm, respectively. In addition, Klencsár et al. [67] also used the co-precipitation method, obtaining magnetite nanopowders with a typical particle size range of 10–20 nm, with different oxidation degrees and a tendency to form agglomerates (200–500 nm in diameter). The authors chose to coat the particles with malic acid to avoid aggregation.

Alternatively, we used PLGA and chitosan as coating materials. These biodegradable polymers are frequent and advantageous options for enhancing the biocompatibility of various nanosystems. Besides, they are particularly appealing for fabricating effective delivery platforms for diverse therapeutics, including antimicrobials [37,68,69,70], antioxidants [71,72,73], anti-inflammatory drugs [16,74,75], and anti-cancer agents [76,77,78,79].

In our study, polymeric encapsulation was noted to increase magnetite-drug particle size by approximately 2 orders of magnitude (from an average diameter of ~15 nm for polymer-free nanoparticles to ~1 μm for the composite spheres). Moreover, polymeric coating resulted in an increase in MIC values for both Gram-positive and Gram-negative bacteria. Nonetheless, despite rising above the results for uncoated magnetite–antibiotic composites, the PLGA/CS/Fe_3_O_4_@STR and PLGA/CS/Fe_3_O_4_@NEO spheres presented low enough MIC values for preserving their antimicrobial character against *S. aureus* and *P. aeruginosa*.

Moreover, a similar reduction in antimicrobial activity by polymer addition was also reported by prior research findings. For example, Hussein-Al-Ali et al. [80] prepared streptomycin-coated chitosan-magnetic nanoparticles (Strep-CS-MNPs) and evaluated their activity against a methicillin-resistant *S. aureus* strain. The authors registered inhibition zones of 17 nm for disk-loaded Strep-CS-MNPs, 21 nm for water-suspended Strep-CS-MNPs, and 32 nm for free streptomycin. The same research group also tested similar Strep-CS-MNPs against *Mycobacterium tuberculosis* and different Gram-positive and Gram-negative bacteria [81]. The study on *Mycobacterium tuberculosis* revealed an MIC of 732 μg/mL for the nanocomposite. In comparison, MIC values of ~4 μg/mL and ~1000 μg/mL were registered for the free antibiotic and free polymer, respectively.

Nonetheless, these results are considered convenient from the biocompatibility point of view, as the polymeric layer could modulate antibiotics release behavior towards minimizing their adverse side effects. This hypothesis is confirmed by the cellular viability studies. Furthermore, the release levels of nitric oxide and lactase dehydrogenase reflect the lack of inflammation and the absence of any toxic effect on the cellular membrane integrity.

## 4. Materials and Methods

After synthesis, the obtained materials were investigated from the compositional, morphological, and biological points of view using X-ray diffraction (XRD), Thermogravimetric Analysis with Differential Scanning Calorimetry (TGA-DSC), Scanning Electron Microscopy (SEM), Transmission Electron Microscopy with Selected Area Electron Diffraction (TEM-SAED), Fourier-Transform Infrared Spectroscopy (FT-IR), cell viability, and antimicrobial tests.

### 4.1. Materials

All reagents required for the synthesis of composite materials, namely anhydrous ferric chloride (FeCl_3_), hydrate ferrous sulfate (FeSO_4_·7H_2_O), ammonia solution (NH3),poly(lactic-co-glycolic acid) (PLGA), neomycin (NEO), streptomycin (STR), chloroform (CHCl_3_), polyvinyl alcohol (PVA), chitosan (CS), and acetic acid were purchased from Sigma Aldrich (Merck Group, Darmstadt, Germany) and used without any further purification.

### 4.2. Synthesis of Fe_3_O_4_@STR and Fe_3_O_4_@NEO Nanoparticles

The antibiotic-functionalized magnetic nanoparticles were prepared using the co-precipitation method, with Fe^2+^ and Fe^3+^ in a 1:2 M ratio, according to Refs. [82,83].

### 4.3. Preparation of Spheres

PLGA/CS/Fe_3_O_4_@STR and PLGA/CS/Fe_3_O_4_@NEO spheres were prepared using a solvent evaporation method [84,85]. Thus, 300 mg PLGA was solubilized in 2 mL CHCl_3_ by sonication. The organic phase was emulsified with a sonicator for 6 min (ON/OFF steps of 5 s and 3 s, limitation temperature of 37 °C) in 14 mL aqueous phase containing 2% (*w/v*) PVA, 20% Fe_3_O_4_@antibiotics (STR/NEO) and CS 1%. After sonication, the emulsion was added in 200 mL deionized water and stirred for 4 h until the complete evaporation of residual CHCl_3_ and then centrifuged at 6000 rpm for 20 min. The obtained spheres were washed four times with ultrapure water, collected by filtration, and finally subjected to freeze drying. Depending on the antibiotic, the resulting systems were denoted as PLGA-CS-Fe_3_O_4_@STR and PLGA-CS-Fe_3_O_4_@NEO.

### 4.4. Characterization Methods

#### 4.4.1. XRD

Phase characterization was realized by XRD using a PANalytical diffractometer, with a CuK α radiation (λ = 1.056 Å), at 15 mA and 30 kV. The experimental determinations were carried out in the Bragg diffraction angle range between 20 and 80°.

#### 4.4.2. SEM

SEM analysis was performed with a Quanta Inspect FEI electron microscope (Thermo Fisher Scientific), using secondary electron beams with energies of 30 keV, on samples coated with a thin gold layer.

#### 4.4.3. TEM

The powder was dispersed in pure ethanol and subjected to a 15-min ultrasonic cleaning treatment. Then, the sample was placed on a carbon-copper grid and left to dry at room temperature. To record the TEM micrographs, a Tecnai^TM^ G2 F30 S-TWIN high-resolution transmission electron microscope from FEI Company (Hillsboro, OR, USA) was used in the transmission mode, at a 300 kV voltage, with point and line resolutions of 2 Å and 1 Å, respectively. Additional crystallographic data were acquired by means of the selected area electron diffraction (SAED) accessory of the same apparatus.

#### 4.4.4. FT-IR

For FT-IR investigations we used a Nicolet 6700 FT-IR spectrometer (Thermo Fisher Scientific). As a result, 32 scans of each sample were realized at room temperature, in a frequency range of 4000–1000 cm^−1^, and a 4 cm^−1^ spectral resolution. The acquired information was recorded by connecting the spectrometer to a unity of data processing using the Omnic Picta 8.2 software (Thermo Fischer Scientific, Waltham, MA, USA).

#### 4.4.5. TGA-DSC

For the thermogravimetric analysis, a reduced powder quantity was placed in an open alumina crucible and heated from room temperature to 900 ℃, at a heating rate of 10 °C/min, under dynamic air atmosphere. An empty alumina crucible was used as a reference. TGA-DSC analyses were performed using a Netzsch STA 449C Jupiter (NETZSCH-Gerätebau GmbH, Selb, Germany).

### 4.5. Biological Characterization

#### 4.5.1. Cell Culture

Human lung cells (MRC-5 cell line, catalog number CCL-171™, from American Type Culture Collection) were used for in vitro tests. The cell culture was maintained at 37 °C, in a humid atmosphere with 5% CO_2_, using Eagle’s Minimum Essential Medium (EMEM) supplemented with 10% fetal bovine serum. To visualize the cells and monitor different growth stages, an Olympus IX71 phase-contrast inverted microscope (Olympus, Tokyo, Japan) was utilized. Prior, the cells were preserved in 95% fetal bovine serum and 5% dimethyl lsulfoxide at −80 °C. Before being placed in culture, they were deiced in a water bath at 37 °C, underwent trypsinization, and were transferred into tubes containing fresh pre-heated medium. The cell pellet necessary for seeding the fibroblasts was obtained by centrifugation for 10 min, at 1500 rotations/min at 18 °C. Next, the supernatant was removed, and the pellet was resuspended in a growth medium, the cells being counted by a Burker-Turk counting chamber. Thus, it was determined that the cell suspension volume had to be added in flasks so that they were seeded at the desired density (3 × 10^4^ cells/cm^2^).

#### 4.5.2. Cell Viability and Toxicity Tests

The cell viability of the magnetite-based nanoparticles and spheres was assessed using the MTT (3-(4,5-dimethylthiazol-2-yl)-2,5-diphenyltetrazolium bromide) viability assay. Cells were incubated for 24 h in a culture medium in the presence of synthesized magnetite-based materials or their absence (as control). After removing the culture medium, the cells were washed with PBS. Then, MTT solution (1 mg/mL) was added, the cells being further incubated at 37 ℃ for two hours in the dark. After its removal, an equal volume of isopropanol was added to solubilize the formazan crystals through pipetting. The spectrophotometric absorbance measurement was realized at a 595 nm wavelength, with a GENios TECAN microplate reader (TECAN, Männedorf, Switzerland).

The viability of the MRC-5 cultures was also visualized with the Live/Dead^®^ Cell Viability Assay (Molecular Probes™, Eugene, OR, USA) on an Olympus IX71 inverted fluorescence microscope. Briefly, after 24 h incubation with nanoparticles, the cells were washed with warm PBS, and incubated with the ethidium-calcein mixture for 30 min at 37 °C. After washing again with PBS, the cells were ready for the imaging procedure.

To establish the nitric oxide concentration (NO) in the culture medium collected after 24 h of incubation with nanoparticles, the Griess reagent, a stoichiometric solution (*v/v*) of 0.1% naphthyl ethylenediamine dihydrochloride and 1% sulphanilamide in 5% H_3_PO_4_, and a NaNO_2_ standard curve were used—the levels of NO increase as a consequence of cytotoxic effects that can trigger inflammation and cell death. Equal volumes of culture supernatants and Griess reagent were mixed, and the absorbance was read at 550 nm using the GENios TECAN microplate reader.

The level of lactate dehydrogenase (LDH) released into the culture media was quantified by using the In Vitro Toxicology Assay Kit, Lactic Dehydrogenase based (TOX7, Sigma-Aldrich, St. Louis, MO, USA). The culture medium was collected after 24 h of cell growth with the tested samples. The absorbance was recorded at 490 nm using a GENios TECAN microplate reader. The statistical analysis was carried out on three replicates per sample by the unpaired Student *t*-test, and differences were considered significant for *p* < 0.05.

#### 4.5.3. Antimicrobial Evaluation

The antibacterial evaluation was performed on bacterial strains of *S. aureus* (ATCC ^®^ 25923) and *P. aeruginosa* (ATCC ^®^ 27853) obtained from the American Type Cell Collection (ATCC, Manassas, VA, USA).

For establishing the MIC of the synthesized nanoparticles and spheres, a quantitative method based on binary serial microdilutions in a liquid medium distributed in 96-well plates was used. In the first well of each row an amount of sample corresponding to a concentration of 1000 μg/mL was added. By means of a micropipette, binary dilutions were performed by a final concentration of 0.05 μg/mL). After realizing the microdilutions, 15 μL of 0.5 McFarland density microbial suspensions was added to each well. The seeded plates were incubated for 24 h at 37 °C and, after incubation, the MIC value for each sample was determined by visual examination as the lowest concentration at which no microbial growth was observed (lack of turbidity). The value was confirmed using a spectrophotometer by reading the absorbance (Optical Density) of microbial culture at 600 nm.

## 5. Conclusions

This study describes a versatile nanostructured and bioactive material to be further developed for anti-infectious therapy. Four magnetite-based nanocomposites were fabricated by co-precipitation and sonication. The obtained nanomaterials showed spherical shape and various sizes, depending on their composition. Antibiotic-functionalized magnetite nanoparticles had a spherical shape, were in the nano range, lacked toxicity towards human cells, and presented low MICs against relevant bacterial strains. Polymeric microspheres successfully encapsulated the nanoparticles, improving their biocompatibility, and maintaining a good antimicrobial effect against *S. aureus* and *P. aeruginosa* bacterial strains. The use of protective polymer structures over streptomycin and neomycin layers has the potential to enrich the applications of these aminoglycoside antibiotics, overcoming their associated toxic side effects. The synthesized materials represent efficient candidates for the controlled delivery of therapeutic agents, promising solutions for preventing and treating ENT-related microbial infections. This study may also serve as an inception point for further research towards designing improved drug delivery systems with magnetic sensitivity, high antibiotic loading, and sustained drug release.

## Figures and Tables

**Table 1:** Thermal analysis data.

Sample	Mass Loss RT-150 °C	Mass Loss 150–450 °C	Residual Mass (%)	Endo	Exo	Estimated Load (%)
Fe_3_O_4_	2.01%	1.85%	95.45%	53.2 °C	565.8 °C	-
Fe_3_O_4_@NEO	5.26%	8.02%	86.46%	106.0 °C	526.2 °C	9.42%
Fe_3_O_4_@STR	2.02%	3.47%	94.08%	84.9 °C	557.7 °C	1.44%

## Data Availability

Available from the authors upon request.
